# Chemical Imaging of Single Anisotropic Polystyrene/Poly (Methacrylate) Microspheres with Complex Hierarchical Architecture

**DOI:** 10.3390/polym13091438

**Published:** 2021-04-29

**Authors:** Alexandra Wagner, Stefanie Wagner, Jan-Erik Bredfeldt, Julia C. Steinbach, Ashutosh Mukherjee, Sandra Kronenberger, Kai Braun, Andreas Kandelbauer, Hermann A. Mayer, Marc Brecht

**Affiliations:** 1 Process Analysis and Technology (PA&T), Reutlingen Research Institute, Reutlingen University, Alteburgstrasse 150, 72762 Reutlingen, Germany; Alexandra.Wagner@Reutlingen-University.de (A.W.); janerik.bredfeldt@googlemail.com (J.-E.B.); Julia.Steinbach@Reutlingen-University.de (J.C.S.); Ashutosh.Mukherjee@Reutlingen-University.de (A.M.); Andreas.Kandelbauer@Reutlingen-University.de (A.K.); 2 Institute of Inorganic Chemistry, University of Tübingen, Auf der Morgenstelle 18, 72076 Tübingen, Germany; stefanie.wagner@uni-tuebingen.de (S.W.); km231@stud.uni-heidelberg.de (S.K.); hermann.mayer@uni-tuebingen.de (H.A.M.); 3 Institute of Physical Chemistry, University of Tübingen, Auf der Morgenstelle 18, 72076 Tübingen, Germany; Braun.Kai@web.de

**Keywords:** anisotropic polymer particles, hierarchical particles, chemical imaging, correlative microscopy, Raman microscopy

## Abstract

Monodisperse polystyrene spheres are functional materials with interesting properties, such as high cohesion strength, strong adsorptivity, and surface reactivity. They have shown a high application value in biomedicine, information engineering, chromatographic fillers, supercapacitor electrode materials, and other fields. To fully understand and tailor particle synthesis, the methods for characterization of their complex 3D morphological features need to be further explored. Here we present a chemical imaging study based on three-dimensional confocal Raman microscopy (3D-CRM), scanning electron microscopy (SEM), focused ion beam (FIB), diffuse reflectance infrared Fourier transform (DRIFT), and nuclear magnetic resonance (NMR) spectroscopy for individual porous swollen polystyrene/poly (glycidyl methacrylate-co-ethylene di-methacrylate) particles. Polystyrene particles were synthesized with different co-existing chemical entities, which could be identified and assigned to distinct regions of the same particle. The porosity was studied by a combination of SEM and FIB. Images of milled particles indicated a comparable porosity on the surface and in the bulk. The combination of standard analytical techniques such as DRIFT and NMR spectroscopies yielded new insights into the inner structure and chemical composition of these particles. This knowledge supports the further development of particle synthesis and the design of new strategies to prepare particles with complex hierarchical architectures.

## 1. Introduction

Modified polystyrene particles are versatile and popular in many scientific fields, e.g., in material sciences, engineering, catalysis, nanomedicine, and biotechnology [1,2,3,4]. The tailored design of particles enables new approaches for imaging and drug delivery [5]. One of the most important approaches is the tuning of particle morphology and topology as well as their charge distribution [4]. The most prominent examples are Janus particles, which are special types of nanoparticles or microparticles whose surfaces have regions of two or more distinctly different physical properties. This unique surface of Janus particles allows different types of chemistry to co-exist on the same particle [6].

The availability of synthetic procedures leading to polystyrene spheres with narrow size distributions and their ability to swell in different media make them ideal templates for the generation of more complex isotropic and anisotropic materials [6,7,8,9,10,11,12,13,14,15]. For this purpose, it is important to control specific particle properties, such as size and size distribution, shape, surface porosity, and chemical composition during synthesis [11].

SEM and bulk-IR as well as solid-state NMR spectroscopy have proven to be the most efficient characterization methods for these particles [16,17,18,19,20,21,22,23,24,25]. The high magnification of SEM images enables precise characterization of their size, shape, and surface properties. Additionally, the combination of SEM and milling with a focused ion beam (FIB) allows insight into their internal structure. However, with these techniques, it is not possible to obtain information about the distribution of chemical components and functionalities across these particles.

The high-resolution imaging technique, 3D confocal Raman microscopy (3D-CRM), is often used in combination with other analytical methods to characterize the chemical and molecular compositions of materials. The correlation of Raman imaging and SEM (RISE), especially, provides information on physical surface properties in combination with molecular compound information of a sample [26,27]. Depth profiles and 3D images can be created with exceptionally good spectral and spatial resolution [28], and the chemical properties can be analyzed with high spatial resolution close to the diffraction limit of light. The 3D-CRM technique is non-invasive, non-destructive, and does not require any complex sample preparation such as staining or labeling [29,30,31,32,33,34]. Since no interference with vibrational bands from water is observed, the particles can be examined directly from aqueous solution.

Here, we present a micro-spectroscopic analysis for the characterization of anisotropic single polystyrene/poly (glycidyl methacrylate-co-ethylene di-methacrylate) particles. The correlation of the results from DRIFT, NMR, SEM, and 3D-CRM enables us to show that particles with Janus-like properties are formed. Using this combination of methods allowed us to observe changes in the particle properties in response to variations in reaction conditions applied during synthesis.

## 2. Materials and Methods

### 2.1. Particle Synthesis

Glycidyl methacrylate (GMA), benzoyl peroxide (BPO), and sodium dodecyl sulfate (SDS) were purchased from abcr GmbH, Karlsruhe, Germany. Ethylene di-methacrylate (EDMA) was obtained from Sigma-Aldrich, Taufkirchen, Germany. Polyvinylpyrrolidone (PVP) and 1-hexanol were supplied from Merck GmbH, Darmstadt, Germany.

We sonicated 1 g of polystyrene and 30 mL of deionized water for 5 min. The suspension was stirred (200 rpm) for 1.5 h at 30 °C and argon was bubbled through the solution. An emulsion of GMA (7 mL), EDMA (7 mL), SDS (0.3 g), PVP (3 g) 25,000 D, 1-hexanol, 0.56 g BPO, and 250 mL of water was prepared by sonicating the mixture (10 min), stirring at RT for 10 min, and bubbling argon through the solution for another 10 min. The emulsion was added to the suspension of the polystyrene particles and stirred for 2 h at 30 °C. The temperature was raised to 70 °C and kept constant for 24 h. The particles were separated and washed with water (3 times) and ethanol (3 times) and dried at 70 °C for 30 min. The volume of 1-hexanol was increased from 7 mL (**S2**) by 14 mL (**S3**) to 21 mL (**S4**).

### 2.2. Particle Characterization

A Hitachi SU8030 (Hitachi High-Technologies Corporation, Tübingen, Germany) was used to obtain SEM images. FIB cuts were prepared with a dual cross-beam FEI Strata DB235 (FEI, Tübingen, Germany). The primary Ga^+^ Ion current was reduced to 50 pA via an aperture. DRIFT spectra were measured with a Bruker Vertex 70 spectrometer (Bruker, Ettlingen, Germany). Solid-state NMR spectra were recorded on a Bruker Avance III-HD spectrometer (Bruker, Ettlingen, Germany) with a wide-bore magnet operating at 300.13 MHz (^1^H) and 75.46 MHz (^13^C). The powdered samples of the respective materials were packed in 4 mm o.d. zirconia rotors; the spinning speed was 10 KHz, the relaxation delay 4 s, and the contact time 2 ms.

For Raman measurements, particle samples were dispersed in ethanol and thoroughly mixed. One droplet (10 µL) of the mixed dispersion was placed on a cleansed sapphire microscope slide and evaporated. Raman spectra and Raman images were obtained using a confocal Raman microscope (WiTec alpha300 RA&S, Ulm, Germany) equipped with an air objective (EC Epiplan-Neofluar DIC M27, 100×, NA = 0.9, Carl Zeiss, Oberkochen, Germany) with a lateral resolution of 360 nm. This system was equipped with a lens-based UHTS 300 spectrometer (WiTec, Ulm, Germany) connected via a 100 µm (NA = 0.12) multi-mode optical fiber and thermoelectric cooled CCD and EMCCD (DU970N-BV, WiTec, Ulm, Germany). All Raman experiments were carried out using a laser of 532 nm with a nominal output power of approx. 39 mW and a grating of 600 lines/mm. Data were processed using Control 5.0 software provided by WITec. All experiments were carried out in ambient conditions. We chose sapphire as a substrate due to its low background signal, which does not interfere with the signal intensity from the region of interest. Additionally, the absorbance signals of sapphire do not overlap with those of the sample, making it an ideal substrate for Raman analysis of these particles.

## 3. Results

Polystyrene particles (**S1**) were synthesized from styrene via a free-radical chain propagation polymerization. Porous poly (glycidyl methacrylate-co-ethylene di-methacrylate) particles **S2**, **S3,** and **S4** were prepared in a seeded swelling polymerization process. In this process, polystyrene particles **S1** were dispersed in water and treated with an emulsion consisting of glycidyl methacrylate (GMA), ethylene di-methacrylate (EDMA), sodium lauryl sulfate (SDS), polyvinyl pyrrolidone (PVP), and 1-hexanol. Benzoyl peroxide was added to start the polymerization. To control the size of the pores, the amount of the porogen (1-hexanol) was gradually increased from 7 mL (**S2**) by 14 mL (**S3**) to 21 mL (**S4**) under otherwise identical reaction conditions.

**Figure 1:**
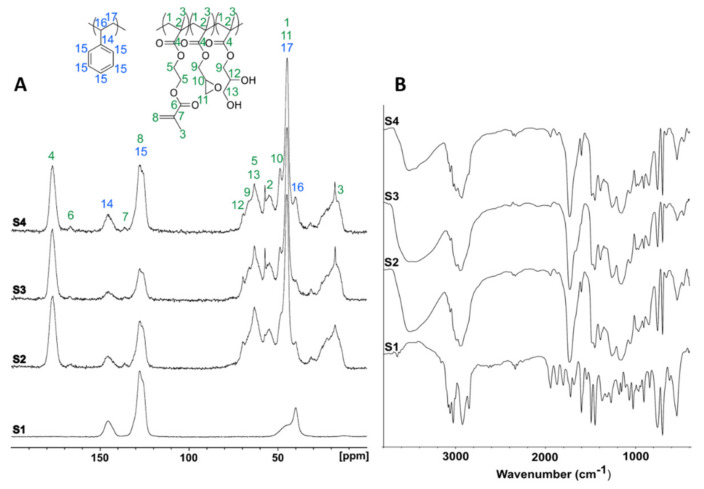
(**A**) Solid-state ^13^C CP/MAS NMR spectra of particles **S1**–**S4** and (**B**) DRIFT spectra of the particles **S1**–**S4**.

Figure 1A shows the solid-state ^13^C CP/MAS NMR spectra of the particles **S1**–**S4**. In the spectra of **S1**, characteristic bands of polystyrene are observed, whereas the spectra of **S2**–**S4** show the characteristic peaks of poly (glycidyl methacrylate-co-ethylene di-methacrylate) polymers as well as those of polystyrene.

The carbon nuclei forming the polymer backbone resonate at 18, 44, and 54 ppm; the oxygen-containing side chains resonate at 63 and 66 ppm, whereas the carbonyl carbon atoms produce to a broad peak at 176 ppm. All samples still contained a considerable amount of polystyrene as indicated by broad peaks in the aromatic region at 127 and 145 ppm. This finding is supported by the comparison of the DRIFT spectrum of polystyrene (**S1**) with those of the particles **S2**–**S4** shown in Figure 1B. All DRIFT spectra of the materials **S2**–**S4** contain absorption bands that are assigned to aromatic C=C at 1600 cm^−1^, C=C–H bending at 700 cm^−1^ and 735 cm^−1^, as well as C=C–H stretching at 3023–3083 cm^−1^. These vibrations of the polystyrene appear along with the characteristic absorptions of poly (glycidyl methacrylate-co-ethylene di-methacrylate) polymers [35]. Characteristic for that polymer is the ester carbonyl (C=O) stretch at around 1725 cm^−1^ and the aliphatic C–H stretch just around 2980 cm^−1^.

**Figure 2:**
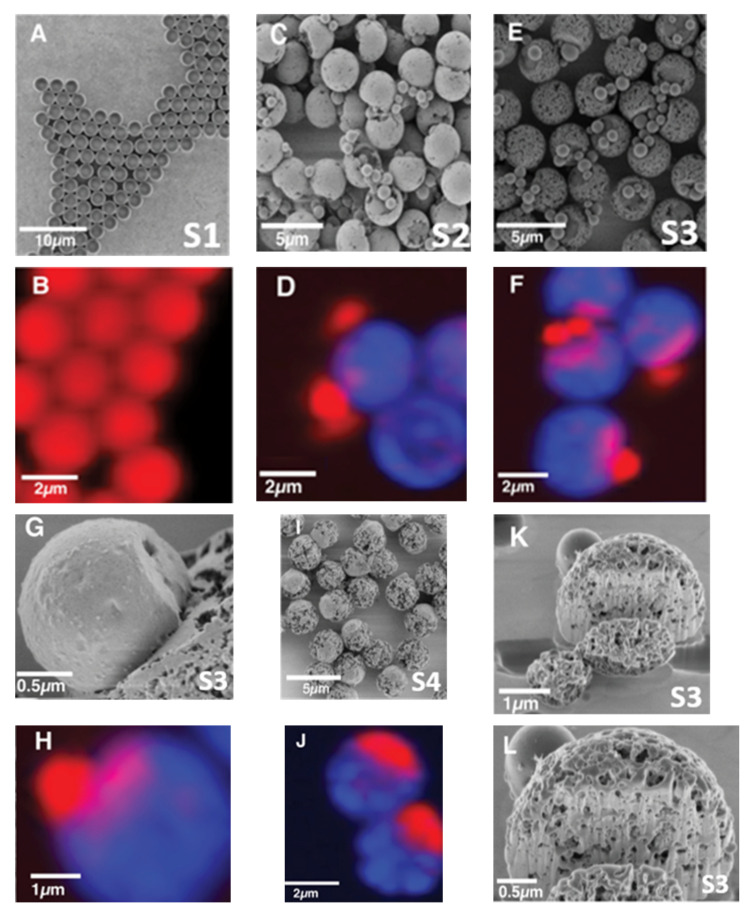
Particles **S1**–**S4** imaged by SEM (**A**,**C**,**E**,**G**,**I**) and the corresponding Raman images (**B**,**D**,**F**,**H**,**J**). A magnified SEM image of **S3** (**G**) and the corresponding Raman image (**H**) are shown. A particle of the sample **S3** milled with Ga^+^ FIB (**K**,**L**). Particles **S1** (**A**,**B**) consist of pure polystyrene. Particles **S2**, **S3**, and **S4** consist of poly (glycidyl methacrylate-co-ethylene di-methacrylate) polymer with an increasing concentration of porogen **S2** (**C**,**D**; 7 mL porogen), **S3** (**E**,**F**; 14 mL porogen), and **S**4 (**I**,**J**; 21 mL porogen).

The surface/bulk porosity, roughness, and chemical distribution were revealed by SEM and 3D-CRM. Figure 2 compares the images of the samples **S1**–**S4** generated by SEM (left column A,C,E,G,I) with those obtained by 3D-CRM (right column B,D,F,H,J). For **S1**, both techniques revealed spherical, highly monodisperse particles. After the swelling of the polystyrene particles **S1** with EDMA, GMA, and 1-hexanol (porogen), larger particles were found with smaller particles attached to them (Figure 2C,E,G,I). The small particles had a more or less spherical shape and a smooth surface; the large particles were dented at one side and are porous. With an increasing amount of porogen, which was in the series **S2** (7 mL), **S3** (14 mL), and **S4** (21 mL), the pore size increased. In the cases of **S2** and **S3**, the small spherical particles were located in the dented area of the large particles (Figure 2C,E,G). Interestingly, the particles **S4** showed non-porous caps in the area of the dent (Figure 2I). To reveal the porosity of the bulk, particles from **S3** were cut through by line milling with a Ga+ FIB (50 pA beam current). The corresponding SEM images (Figure 2K,L) demonstrate that the porosity inside the main particle is comparable to that on its surface.

The 3D-CRM images of the samples **S1**–**S4** shown in Figure 2B,D,F,H,J reproduced the size and the shape of the particles as shown in the corresponding SEM images. Due to the poor resolution of the optical light-microscopy compared with SEM, the porosity and surface morphology can only be assumed. However, the advantage of 3D-CRM is that the chemical composition and distribution of the sample can be deduced by the fingerprint Raman spectrum at different positions on the particles.

**Figure 3:**
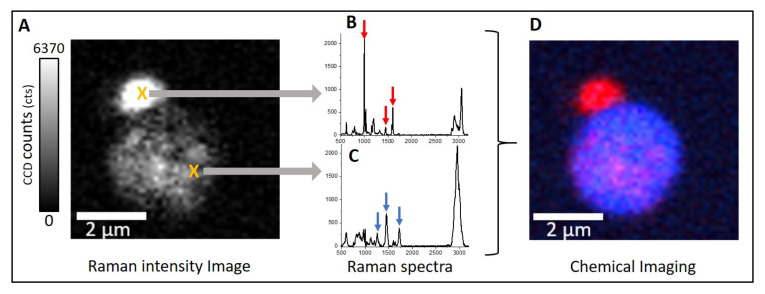
(**A**) Raman intensity image of a particle **S3**. (**B**,**C**) Raman spectra recorded in the range from 500 to 3200 cm^−1^ at the locations marked with X. Characteristic Raman signals are marked with red and blue arrows (see text for details). (**D**) The Raman image is based on the assignment of spectra in each pixel. Pixels dominated by spectrum (**B**,**C**) are color-coded with red and blue, respectively.

The assignment of the Raman peaks to specific vibration bands and the resulting color coding of the images are shown in Figure 3. Figure 3A shows a two-dimensional scan of one particle. Here, the integral intensity of Raman scattering in the range of 350–3200 cm^−1^ is used. The particle is distinguishable from the background. In the region of the attached small particle, the observed intensity is significantly higher compared with the main particle. Figure 3B,C shows Raman spectra recorded at the positions marked by X. The Raman spectrum recorded at the position of the small particle can be assigned to polystyrene due to the characteristic Raman peaks at 1002, 1603, and 1448 cm^−1^ (red arrows) [35,36,37]. The Raman spectrum recorded at the main particle can be assigned to poly (glycidyl methacrylate-co-ethylene di-methacrylate) polymer due to the Raman peak at 1723 cm^−1^ [35]. The peaks at 1454 and 1259 cm^−1^ (blue arrows) can be assigned to ketones and epoxides from EDMA and GMA, respectively [35,38,39].

A principal component analysis (PCA) of all Raman spectra yielded two principal components explaining 95% of the total data. Based on this number of relevant components, the data were analyzed with Control 5.0 data processing software provided by WITec using an inbuilt PCA. The components were assigned to colors (blue, red) for better visualization. Regions dominated by the polystyrene were assigned the color red, while those dominated by EDMA/GMA were assigned the color blue; the resulting image is depicted in Figure 3D. Consequently, the small particles attached to the larger particles could be assigned to polystyrene and the large particles to EDMA/GMA. Based on this assignment, all 3D-CRM images in Figure 2 are color-coded following the same procedure.

## 4. Discussion

A correlation between NMR, DRIFT, SEM, and 3D-CRM is presented in this study of polystyrene particles. With the combination of these techniques, the swelling of polystyrene/poly (glycidyl methacrylate-co-ethylene di-methacrylate) particles depending on the concentration of the porogen (1-hexanol) was studied. The ^13^C CP/MAS NMR spectra (Figure 1A) show that, in all swollen particles, polystyrene and poly (glycidyl methacrylate-co-ethylene di-methacrylate) are present. These results are consistent with the results of the DRIFT measurements (Figure 1B).

The first indications of a deviation from uniformity/sphericity of the particles are found in the SEM images (Figure 2). The SEM images of the particles from the synthesis using 7 mL (**S2**) (Figure 2C) and 14 mL (**S3**) (Figure 2E) porogen show different types of particles. They clearly show small- and large-sized particles. The large particles show a dented area where the small particles are attached. The relative amount of small particles in relation to the larger ones varies within the sample. SEM revealed that the surface roughness of the small particles is lower than that of the large particles. When 21 mL (**S4**) (Figure 2I) of porogen was used, no dented areas or small particles were observed. The **S4** particles were almost spherical, with a flat nonporous cap in the region wherein the dented areas in the other particles were observed. This reduced roughness is comparable to the roughness that was found for small particles (**S2** and **S3**) at lower porogen concentrations.

The porosity of the particles on the surface and in the bulk was further investigated. For this, the particles were milled with FIB. SEM images of a milled particle **S3** (14 mL porogen) are shown in Figure 2K,L. The FIB image indicates a comparable porosity throughout the particle. Milling of the smaller particles was unsuccessful.

Next, the chemical composition of individual particles was studied in detail with 3D-CRM. The analysis of the Raman spectra enables an assignment of the observed vibrational bands to molecule specific vibrations. The spectra recorded at the position of the small particles are dominated by vibrations assigned to polystyrene, whereas the spectra recorded at the large particles show bands assigned to poly (glycidyl methacrylate-co-ethylene di-methacrylate). The CRM images suggest that polystyrene is extruded from the forming EDMA/GMA polymer network during polymerization. When the porogen concentration was increased to 21 mL (**S4**) (Figure 2I,J), the extrusion of polystyrene remained incomplete, and the extruded polystyrene formed an adherent cap on the dented areas of the large particles.

**Scheme 1:**
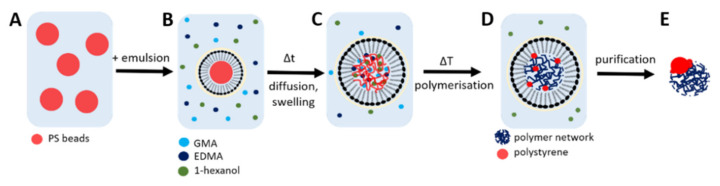
(**A**,**B**) Suspension of monodisperse micron-sized polystyrene beads in water mixed with an emulsion consisting of GMA, EDMA, SDS, PVP, BPO, and 1-hexanol (in adequate quantities for **S2**, **S3**, and **S4**). For clarity, BPO (radical starter) is not included in the scheme. Hydrophobic attractions between PS and SDS are responsible for the formation of micelles. Water-soluble PVP stabilizes the micelles from the outside. (**C**) The particles swell due to the diffusion of GMA, EDMA, 1-hexanol, and BPO into the bead. (**D**) Upon polymerization, both methacrylates form the porous poly (glycidyl methacrylate-ethylene dimethacrylate) network with 1-hexanol as an inert solvent. (**E**) At a certain point, the micelles rupture and release a porous particle.

A presumed mechanism for the formation of these particles is shown in Scheme 1.

Pore formation in particles depends on the solubility of porogen and the seed particle. Materials with similar solubility parameters have a high affinity to each other. Notably, 1-hexanol has a poor theoretical solubility with polystyrene and poly (glycidyl methacrylate-co-ethylene di-methacrylate). As polymerization starts, phase separation occurs due to insufficient solubility and 1-hexanol is displaced as an inert molecule, leaving the pore structure in the particle. In the magnified SEM image (Figure 2G), it appears that polystyrene is squeezed out of the particle. This can be also explained by solubility effects. Due to a rather good solubility in GMA and EDMA, the polystyrene bead is dissolved into individual polymer chains of high mobility and flexibility [40,41,42]. Upon GMA and EDMA polymerization, phase separation occurs again, and polystyrene becomes insoluble in the three-dimensional network and is squeezed out.

The syntheses described here yield particles with variable size, shape, and porosity. This is achieved by changing only one parameter, which is the amount of porogen added during synthesis. In chemistry, managing critical parameters precisely plays a crucial role in the final properties of the particles [11]. The 3D-CRM results revealed that these particles show a Janus particle behavior not only in terms of their morphology, but also in terms of their chemical composition. Both properties are controllable by the concentration of the porogen. We also showed that 3D-CRM is a useful tool for analyzing and characterizing inhomogeneous particles in a spatially resolved and three-dimensional way. Thus, the synthesis can be adjusted accordingly to achieve particles of defined 3D morphology and controlled chemical composition. Potential applications of these particles include as template molecules for large porous silica microspheres for protein separation, drug delivery systems, microcapsule systems for extrinsic self-healing applications, or, for instance, as templating materials used in energy storage applications [18,43,44,45,46,47,48,49,50,51].

## 5. Conclusions

This study shows that the combination of NMR, DRIFT, SEM, and 3D-CRM is well-suited for controlled synthesis of polymer particles. Synthesized particles with varying proportions of porogen were analyzed with the aforementioned methods at each step of synthesis development. This provides direct insight into the development of the synthesis at every step. Both solid-state **^13^**C CP/MAS NMR and DRIFT spectra showed that all particles (**S1**, **S2**, **S3**, and **S4**) contain a significant amount of polystyrene. Swollen particles (**S2**, **S3** and **S4**) showed both polystyrene and other constituent byproducts as a result of synthesis. Interpretation of the morphology and the chemical composition of the particles was facilitated by the combined use of SEM and 3D-CRM. This approach revealed the distribution of the chemical entities across the particles. We found that 3D-CRM is indispensable for the assignment of the different chemical entities to the morphological features on the particles, and distribution of chemical species can be visualized using PCA. Similar problems are often analyzed with fluorescence microscopy; however, this requires the addition of staining chemicals or labeling steps [29,30,31]. For the selection of appropriate labels, making assumptions about the resulting particles is necessary. Consequently, the selection of the labels influences the information accessible by the measurement and may bias the result. In contrast, the marker-free approach presented here minimizes sample alteration and eliminates the need for an additional step, since no labeling is necessary for 3D-CRM measurements and the Raman spectra provide a detailed insight into the chemical composition.

## Data Availability

The data presented in this study are available on request from the corresponding author.
